# Crystal structure of 2-diazo­imidazole-4,5-dicarbo­nitrile

**DOI:** 10.1107/S2056989015010944

**Published:** 2015-06-17

**Authors:** Damon A. Parrish, Stephanie Kramer, G. Kenneth Windler, David E. Chavez, Philip W. Leonard

**Affiliations:** a CBMSE, Code 6910, Naval Research Laboratory, Washington, DC 20375, USA; b PO Box 1663 MS C920, Los Alamos National Laboratory, Los Alamos, NM 87545, USA

**Keywords:** crystal structure, diazo, imidazole, carbo­nitrile

## Abstract

In the title compound, C_5_N_6_, all the atoms are approximately coplanar. In the crystal, mol­ecules are packed with short contact distances of 2.885 (2) (between the diazo N atom connected to the ring and a cyano N atom on a neighboring mol­ecule) and 3.012 (2) Å (between the terminal diazo N atom and an N atom of a neighboring imidazole ring).

## Related literature   

For synthesis of the title compound, see: Lu & Just (2001 ▸); Sheppard & Webster (1973 ▸). Few diazo-containing mol­ecules have been isolated, and of these, only a small number have been examined by X-ray diffraction, see: Daidone *et al.* (2005 ▸); Dippold *et al.* (2012 ▸). The majority of these compounds are found as diazo­nium ions, rather than the neutral diazo species, see: Bugg *et al.* (1964 ▸).
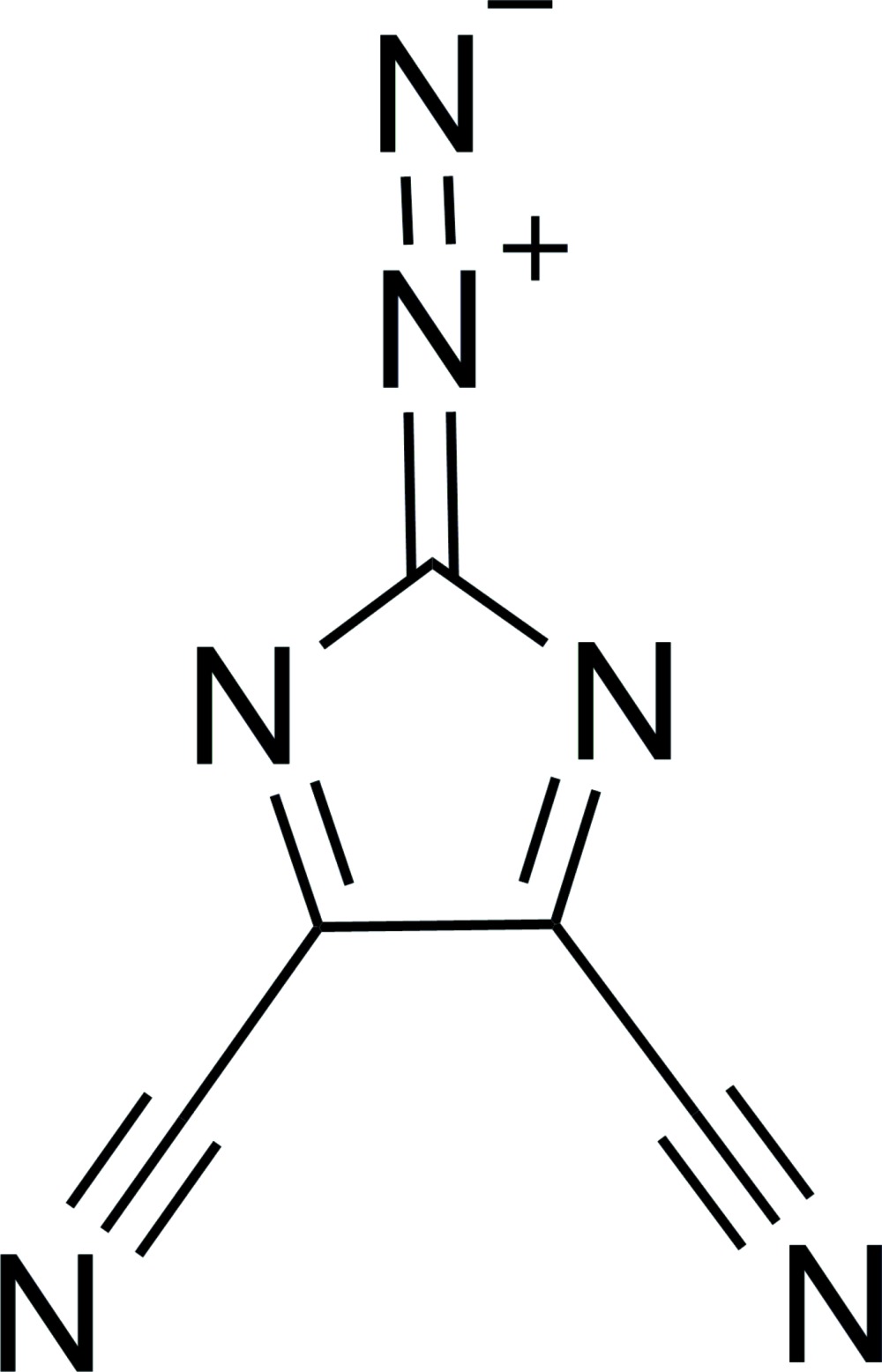


## Experimental   

### Crystal data  

C_5_N_6_
*M* *_r_* = 144.11
Trigonal, 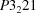

*a* = 8.0746 (3) Å
*c* = 16.7315 (6) Å
*V* = 944.73 (8) Å^3^
*Z* = 6
Mo *K*α radiation
μ = 0.11 mm^−1^
*T* = 150 K
0.37 × 0.30 × 0.08 mm

### Data collection  

Bruker SMART APEXII CCD diffractometer
Absorption correction: multi-scan (*SADABS*; Bruker, 2008 ▸) *T*_min_ = 0.930, *T*_max_ = 0.991
9178 measured reflections
1289 independent reflections
1269 reflections with *I* > 2σ(*I*)
*R*_int_ = 0.019

### Refinement  

*R*[*F* ^2^ > 2σ(*F* ^2^)] = 0.021
*wR*(*F* ^2^) = 0.058
*S* = 1.09
1289 reflections
100 parameters
Δρ_max_ = 0.14 e Å^−3^
Δρ_min_ = −0.11 e Å^−3^

### 

Data collection: *APEX2* (Bruker, 2009 ▸); cell refinement: *APEX2* and *SAINT* (Bruker, 2009 ▸); data reduction: *SAINT* (Bruker, 2009 ▸) and *XPREP* (Bruker, 2008 ▸); program(s) used to solve structure: *SHELXTL* (Sheldrick, 2008 ▸); program(s) used to refine structure: *SHELXTL*; molecular graphics: *ORTEP-3 for Windows* (Farrugia, 2012 ▸) and *Mercury* (Macrae *et al.*, 2008 ▸); software used to prepare material for publication: *CHEMDRAW Ultra* (Cambridge Soft, 2014 ▸).

## Supplementary Material

Crystal structure: contains datablock(s) global, I. DOI: 10.1107/S2056989015010944/cq2016sup1.cif

Structure factors: contains datablock(s) I. DOI: 10.1107/S2056989015010944/cq2016Isup2.hkl

Supporting information file. DOI: 10.1107/S2056989015010944/cq2016Isup3.cdx

Supporting information file. DOI: 10.1107/S2056989015010944/cq2016Isup4.cml

**Fig. 1.**
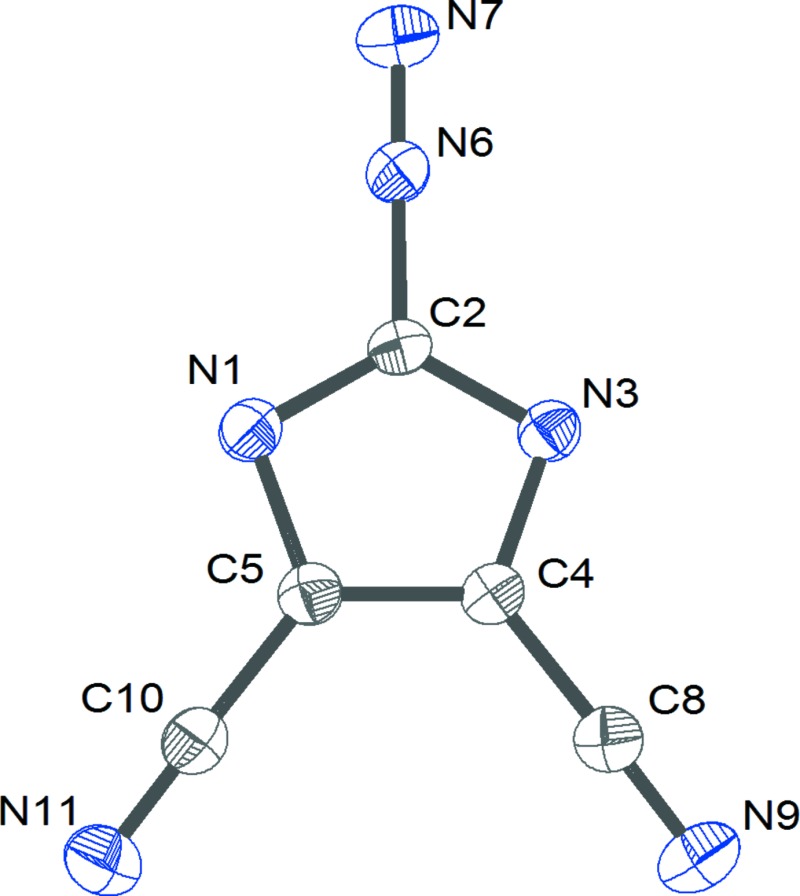
The molecular structure of the title compound (displacement ellipsoids are drawn at the 50% probability level).

**Fig. 2.**
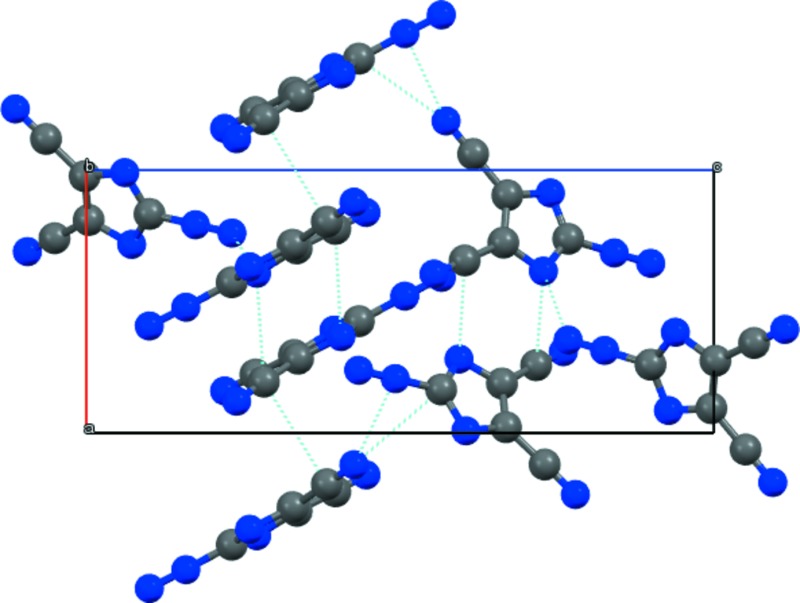
A packing diagram of the title compound viewed along the b axis. Close contacts are represented by dashed lines.

. DOI: 10.1107/S2056989015010944/cq2016fig1.tif
The mol­ecular structure of the title compound (displacement ellipsoids are drawn at the 50% probability level).

b . DOI: 10.1107/S2056989015010944/cq2016fig2.tif
A packing diagram of the title compound viewed along the *b* axis. Close contacts are represented by dashed lines.

CCDC reference: 1056377

Additional supporting information:  crystallographic information; 3D view; checkCIF report

## supplementary crystallographic information

### S1. Comment

Diazo moieties are ubiquitious in organic chemistry due to their ability to act as as outstanding leaving groups in substitution reactions. This same reactivity also leads to their instability, and most are prone to decomposition, often violently. For this reason, few diazo-containing molecules have been isolated, and of these, only a small number have been examined by X-ray diffraction [Daidone *et al.* (2005), Dippold *et al.* (2012)]. The majority of these compounds are found as diazonium ions, rather than the neutral diazo species [Bugg *et al.* (1964)]. In contrast, the title compound has a neutral diazo moiety on C2 and is unusual in that it contains only carbon and nitrogen.

### S2. Experimental

Caution! This compound can explode from slight friction, impact, or thermal shock. Although the explosion is not powerful, it is recommended that this material is only prepared in less than 5 gram quantities, handled wet, and not confined in any way.

The title compound was prepared by the literature method (Lu and Just, 2001). Crystals were obtained by slow evaporation of a dilute aqueous solution of the title compound.

### S3. Refinement

The Flack parameter initially refined to 0.3 (5). Before the final refinement cycle, this parameter was reset to zero and the Friedel pairs merged.

### Crystal data

**Table:**

C_5_N_6_	*D*_x_ = 1.520 Mg m^−^^3^
*M**_r_* = 144.11	Melting point: 413 K (expl.) K
Trigonal, *P*3_2_21	Mo *K*α radiation, λ = 0.71073 Å
*a* = 8.0746 (3) Å	µ = 0.11 mm^−^^1^
*c* = 16.7315 (6) Å	*T* = 150 K
*V* = 944.73 (8) Å^3^	Plate, purple
*Z* = 6	0.37 × 0.30 × 0.08 mm
*F*(000) = 432	

### Data collection

**Table:**

Bruker SMART APEXII CCD diffractometer	1269 reflections with *I* > 2σ(*I*)
Radiation source: fine focus sealed tube	*R*_int_ = 0.019
ω scans	θ_max_ = 26.4°, θ_min_ = 3.2°
Absorption correction: multi-scan (*SADABS*; Bruker, 2008)	*h* = −10→10
*T*_min_ = 0.930, *T*_max_ = 0.991	*k* = −10→10
9178 measured reflections	*l* = −20→20
1289 independent reflections	

### Refinement

**Table:**

Refinement on *F*^2^	100 parameters
Least-squares matrix: full	0 restraints
*R*[*F*^2^ > 2σ(*F*^2^)] = 0.021	*w* = 1/[σ^2^(*F*_o_^2^) + (0.0333*P*)^2^ + 0.1041*P*] where *P* = (*F*_o_^2^ + 2*F*_c_^2^)/3
*wR*(*F*^2^) = 0.058	(Δ/σ)_max_ = 0.001
*S* = 1.09	Δρ_max_ = 0.14 e Å^−^^3^
1289 reflections	Δρ_min_ = −0.11 e Å^−^^3^

### Special details

**Table:**

Geometry. All e.s.d.'s (except the e.s.d. in the dihedral angle between two l.s. planes) are estimated using the full covariance matrix. The cell e.s.d.'s are taken into account individually in the estimation of e.s.d.'s in distances, angles and torsion angles; correlations between e.s.d.'s in cell parameters are only used when they are defined by crystal symmetry. An approximate (isotropic) treatment of cell e.s.d.'s is used for estimating e.s.d.'s involving l.s. planes.
Refinement. Refinement of *F*^2^ against ALL reflections. The weighted *R*-factor *wR* and goodness of fit *S* are based on *F*^2^, conventional *R*-factors *R* are based on *F*, with *F* set to zero for negative *F*^2^. The threshold expression of *F*^2^ > σ(*F*^2^) is used only for calculating *R*-factors(gt) *etc*. and is not relevant to the choice of reflections for refinement. *R*-factors based on *F*^2^ are statistically about twice as large as those based on *F*, and *R*- factors based on ALL data will be even larger.

### Fractional atomic coordinates and isotropic or equivalent isotropic displacement parameters (Å^2^)

**Table:**

	*x*	*y*	*z*	*U*_iso_*/*U*_eq_	
N1	0.61645 (16)	0.00825 (16)	0.93835 (6)	0.0254 (3)	
C2	0.7396 (2)	0.16549 (19)	0.89882 (7)	0.0226 (3)	
N3	0.91448 (16)	0.28157 (17)	0.92732 (6)	0.0239 (3)	
C4	0.90565 (19)	0.18858 (19)	0.99570 (7)	0.0225 (3)	
C5	0.72502 (19)	0.02197 (19)	1.00215 (7)	0.0240 (3)	
N6	0.68567 (16)	0.20682 (16)	0.82574 (6)	0.0238 (3)	
N7	0.6461 (2)	0.23873 (19)	0.76756 (7)	0.0329 (3)	
C8	1.0629 (2)	0.2582 (2)	1.05016 (7)	0.0258 (3)	
N9	1.18839 (19)	0.3094 (2)	1.09341 (7)	0.0351 (3)	
C10	0.6572 (2)	−0.1202 (2)	1.06366 (8)	0.0299 (3)	
N11	0.6055 (2)	−0.2345 (2)	1.11254 (8)	0.0441 (4)	

### Atomic displacement parameters (Å^2^)

**Table:**

	*U*^11^	*U*^22^	*U*^33^	*U*^12^	*U*^13^	*U*^23^
N1	0.0235 (6)	0.0266 (6)	0.0250 (5)	0.0117 (5)	−0.0016 (4)	−0.0005 (5)
C2	0.0241 (6)	0.0255 (6)	0.0200 (5)	0.0138 (5)	−0.0021 (5)	−0.0011 (5)
N3	0.0234 (6)	0.0258 (6)	0.0226 (5)	0.0125 (5)	−0.0006 (4)	−0.0012 (4)
C4	0.0223 (6)	0.0251 (6)	0.0223 (6)	0.0135 (5)	−0.0002 (5)	−0.0022 (5)
C5	0.0237 (6)	0.0266 (6)	0.0237 (6)	0.0140 (6)	0.0002 (4)	−0.0005 (5)
N6	0.0231 (6)	0.0256 (6)	0.0244 (5)	0.0136 (5)	−0.0005 (4)	−0.0020 (4)
N7	0.0370 (7)	0.0425 (8)	0.0282 (6)	0.0265 (6)	−0.0027 (5)	−0.0003 (5)
C8	0.0266 (7)	0.0298 (7)	0.0239 (6)	0.0161 (6)	0.0024 (5)	−0.0005 (5)
N9	0.0310 (7)	0.0467 (8)	0.0306 (6)	0.0216 (6)	−0.0070 (5)	−0.0064 (5)
C10	0.0240 (7)	0.0324 (7)	0.0314 (7)	0.0127 (6)	−0.0020 (5)	0.0025 (6)
N11	0.0362 (7)	0.0456 (8)	0.0451 (8)	0.0164 (7)	−0.0007 (6)	0.0179 (6)

### Geometric parameters (Å, º)

**Table:**

N1—C2	1.3327 (18)	C4—C8	1.4297 (18)
N1—C5	1.3502 (16)	C5—C10	1.4314 (18)
C2—N3	1.3325 (18)	N6—N7	1.0946 (15)
C2—N6	1.3936 (15)	C8—N9	1.1415 (19)
N3—C4	1.3507 (17)	C10—N11	1.144 (2)
C4—C5	1.4094 (18)		
			
C2—N1—C5	99.75 (11)	C5—C4—C8	128.18 (12)
N3—C2—N1	121.08 (11)	N1—C5—C4	109.72 (11)
N3—C2—N6	119.59 (12)	N1—C5—C10	122.03 (12)
N1—C2—N6	119.33 (12)	C4—C5—C10	128.23 (12)
C2—N3—C4	99.74 (11)	N7—N6—C2	178.50 (12)
N3—C4—C5	109.70 (11)	N9—C8—C4	178.23 (16)
N3—C4—C8	122.12 (13)	N11—C10—C5	178.82 (17)
			
C5—N1—C2—N3	−0.01 (16)	C2—N1—C5—C4	0.36 (14)
C5—N1—C2—N6	178.72 (11)	C2—N1—C5—C10	−178.37 (13)
N1—C2—N3—C4	−0.34 (16)	N3—C4—C5—N1	−0.61 (16)
N6—C2—N3—C4	−179.07 (11)	C8—C4—C5—N1	179.57 (12)
C2—N3—C4—C5	0.53 (14)	N3—C4—C5—C10	178.02 (13)
C2—N3—C4—C8	−179.64 (12)	C8—C4—C5—C10	−1.8 (2)
