# Supplementary figures and images for: Crystal structure of 2-diazo­imidazole-4,5-dicarbo­nitrile
Source: Acta Crystallogr E Crystallogr Commun. 2015 Jun 17;71(Pt 7):o491. doi: 10.1107/S2056989015010944 (PMC4518995; doi:10.1107/S2056989015010944)

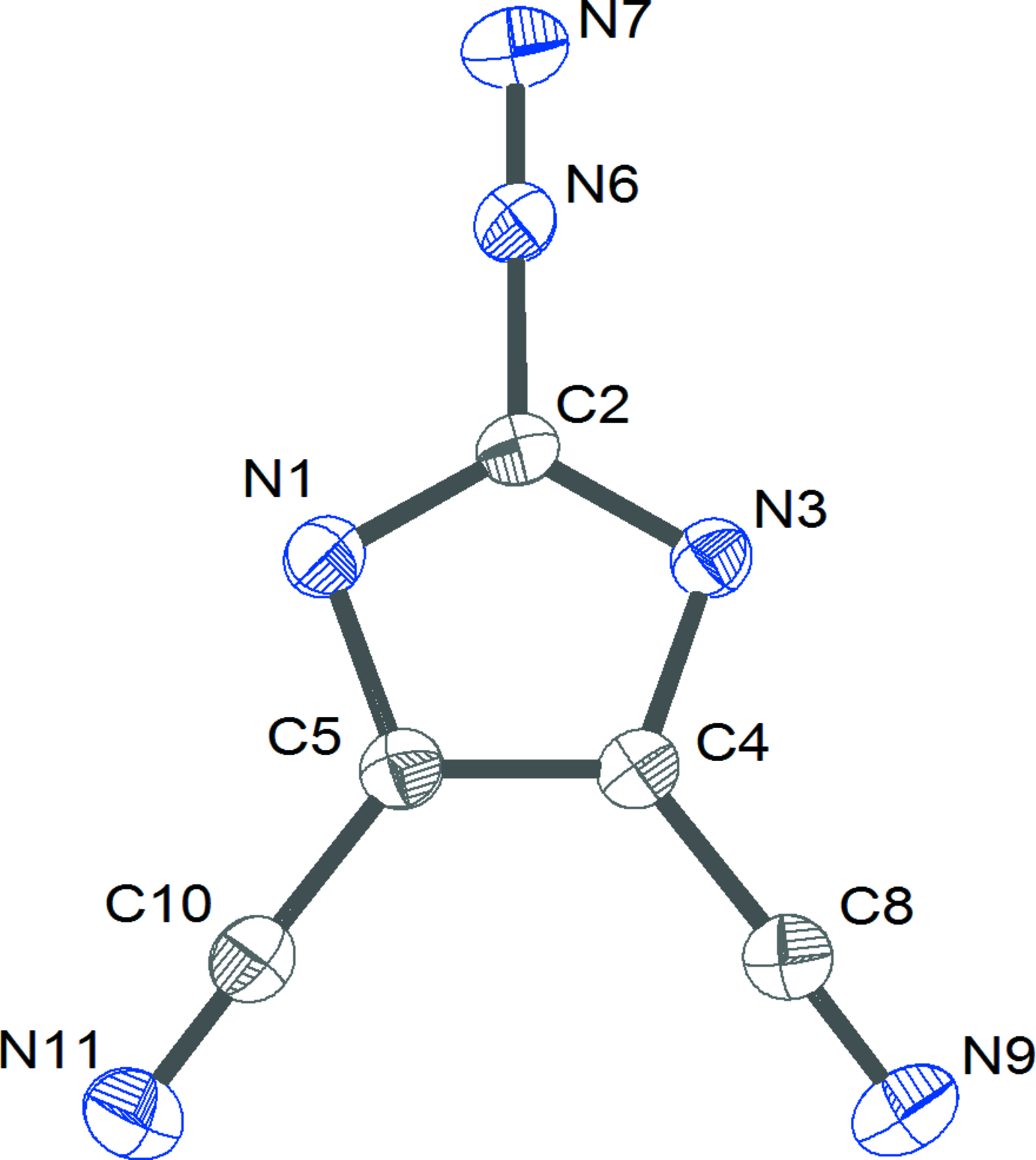

Supplement: Supplementary file 5 [file e-71-0o491-fig1.tif]

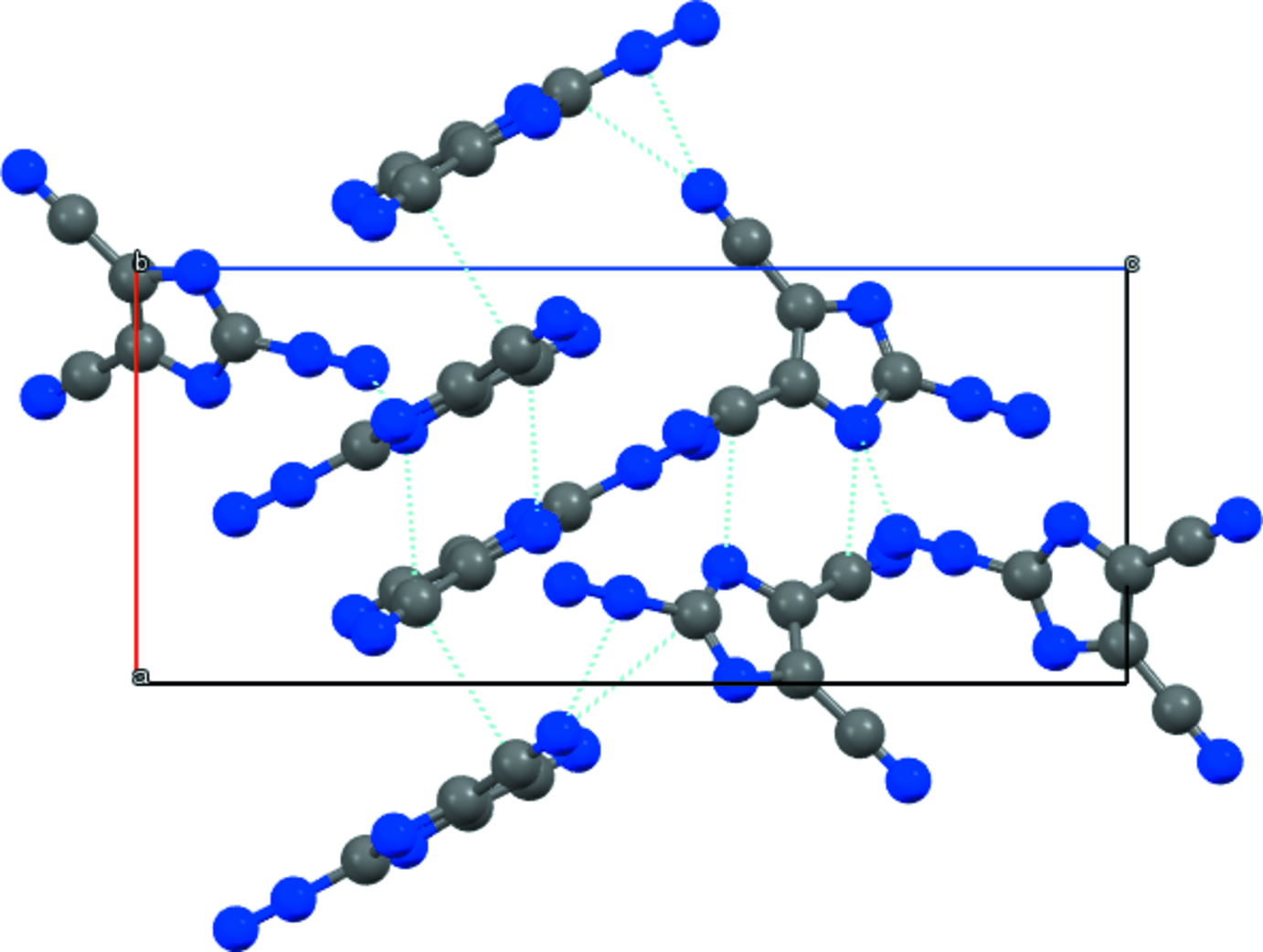

Supplement: Supplementary file 6 [file e-71-0o491-fig2.tif]
